# Two Cases of *γ*-Heavy Chain Disease and a Review of the Literature

**DOI:** 10.1155/2018/4832619

**Published:** 2018-08-12

**Authors:** I. Ramasamy, Z. Rudzki

**Affiliations:** ^1^Department of Biochemistry, Worcester Royal Hospital, Worcester, UK; ^2^Heart of England NHS Trust, Birmingham, UK

## Abstract

Gamma heavy chain disease (*γ*-HCD) is a rare lymphoproliferative disorder characterised by the production of a truncated immunoglobulin heavy chain. Fewer than 200 cases have been reported in the literature. In some cases, *γ*-HCD occurs with other lymphoid neoplasms. This study reports clinical, biochemical, haematological, and histological findings in two cases of *γ*-HCD. We describe newer biochemical diagnostic tools (HevyLite measurement, capillary electrophoresis, and immunotyping) that can aid in the characterisation of *γ*-HCD. The first case is an 88-year-old woman with *γ*-HCD. The second case is an 81-year-old woman who developed *γ*-HCD during treatment for Waldenstrom's macroglobulinemia. In the second patient, histopathology identified a separate clone responsible for the secretion of the gamma heavy chain. Studies on the clonal evolution of the disease may provide insight into therapeutic implications and the genomic complexity of the disease.

## 1. Introduction

Gamma heavy chain disease (*γ*-HCD) is also called Franklin's disease, named after the author of the first report in 1964. The disease is defined as a neoplasm of lymphocytes, plasmacytoid lymphocytes, and plasma cells characterised by the production of an abnormally truncated gamma heavy chain protein that lacks associated light chains. Since Franklin first reported the case, fewer than 200 cases have been described in the literature. A wide variety of disorders have been associated with *γ*-HCD. In some cases, *γ*-HCD occurs with other lymphoid neoplasms or with a history of autoimmune disorder. *γ*-HCD shows a wide clinical spectrum ranging from completely asymptomatic to progressively malignant forms.

Normal heavy chains not associated with light chains have not been detected in serum of healthy individuals. In *γ*-HCD abnormally short truncated heavy chains can be isolated from patient's serum without associated light chains. In several instances where the gene encoding the shortened heavy chain gene has been characterised, the truncation originated from deletions and/or insertions within the rearranged variable region genes (V) that in addition harbor somatic point mutations. One possibility is that several types of oncogene translocations happen as a by-product of somatic hypermutation [1]. It has been established the *γ*-HCD immunoglobulins are the products of aberrant biosynthesis, for example, deletions, splice site correction, and amino-terminal proteolysis [2]. Structural analysis of the defective gamma heavy chain in 23 patients showed some common features. In most cases, the variable region sequence was short and interrupted by large deletions, usually including the C_H_1 domain. Normal sequence began at the hinge region or at the C_H_2 domain [3].

Genetic subtypes of multiple myeloma (MM) have been identified which have different biological features and heterogeneity in clinical outcomes [4]. New gene sequencing techniques have yielded new insights into the pathogenesis and evolution of the disease and potential individualised treatment by novel targeted approaches [5].

We report two cases of *γ*-HCD: one case in a patient with lymphadenopathy and a second case in a patient with a history of Waldenstrom's macroglobulinemia (WM). Further, we review previous case series on *γ*-HCD. These two cases were diagnosed using the techniques of capillary zone electrophoresis, immunofixation, and “HevyLite” measurement. Use of newer commercially available techniques simplified the diagnosis of *γ*-HCD. In one patient, there was no evidence of previous chemotherapy. The second patient developed *γ*-HCD during treatment for WM.

## 2. Case Report

### 2.1. Case 1

An 88-year-old woman presented with a change in the bowel habit. A colonoscopy showed some diverticular disease. The CT scan showed splenomegaly and some lymphadenopathy particularly in the region of the splenic hilum. The liver, kidney, pancreas, and adrenals were normal. She had a past history of osteopenia, type II diabetes, and fragility fracture. She was taking vitamin B12, vitamin D, and bisphosphonates. There was no history of sweating, weight loss, bruising, or recent infections. Her biochemistry and hematology at diagnosis and 3 months after diagnosis are summarised in Tables 1 and 2.

**Figure 1:**
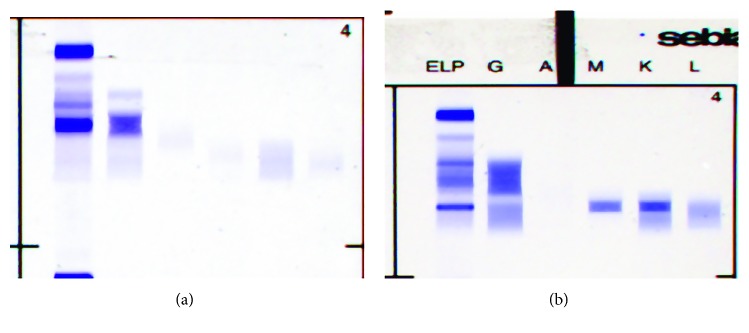
Serum electrophoresis and immunofixation. (a) Case 1: immunofixation shows staining in the gamma region, without a corresponding stain in the light chain region. Two bands are detected in the gamma region, a major and a second minor band. (b) Case 2: immunofixation shows a broad stain in the alpha-beta region, without a corresponding region in the light chain. Immunofixation further identifies an IgMkappa paraprotein. ELP = protein electrophoresis; G: immunofixation with anti-gamma chain; identifying gamma heavy chain; A: anti-alpha chain immunofixation; M: anti-mu chain immunofixation; K: anti-kappa chain immunofixation; L: anti-lambda chain immunofixation.

**Figure 2:**
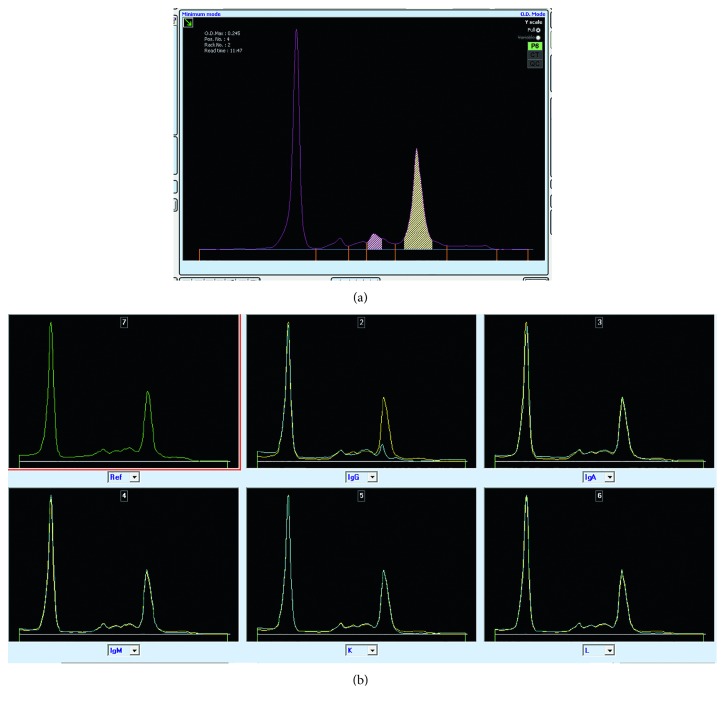
Capillary zone electrophoresis and immunotyping of Case 1. (a) Capillary zone electrophoresis showing a peak in the beta1 region and a minor peak in the alpha region. (b) Immunotyping showing a subtraction by the anti-IgG antibody, without a corresponding region in the light chain region, identifying a gamma chain peak. Ref: protein electrophoresis. IgG: immunosubtraction with anti-IgG antibody; IgA: immunosubtraction with anti-IgA antibody; IgM: immunosubtraction with anti-IgM antibody; K: immunosubtraction with anti-kappa antibody; L: immunosubtraction with anti-lambda antibody.

**Figure 3:**
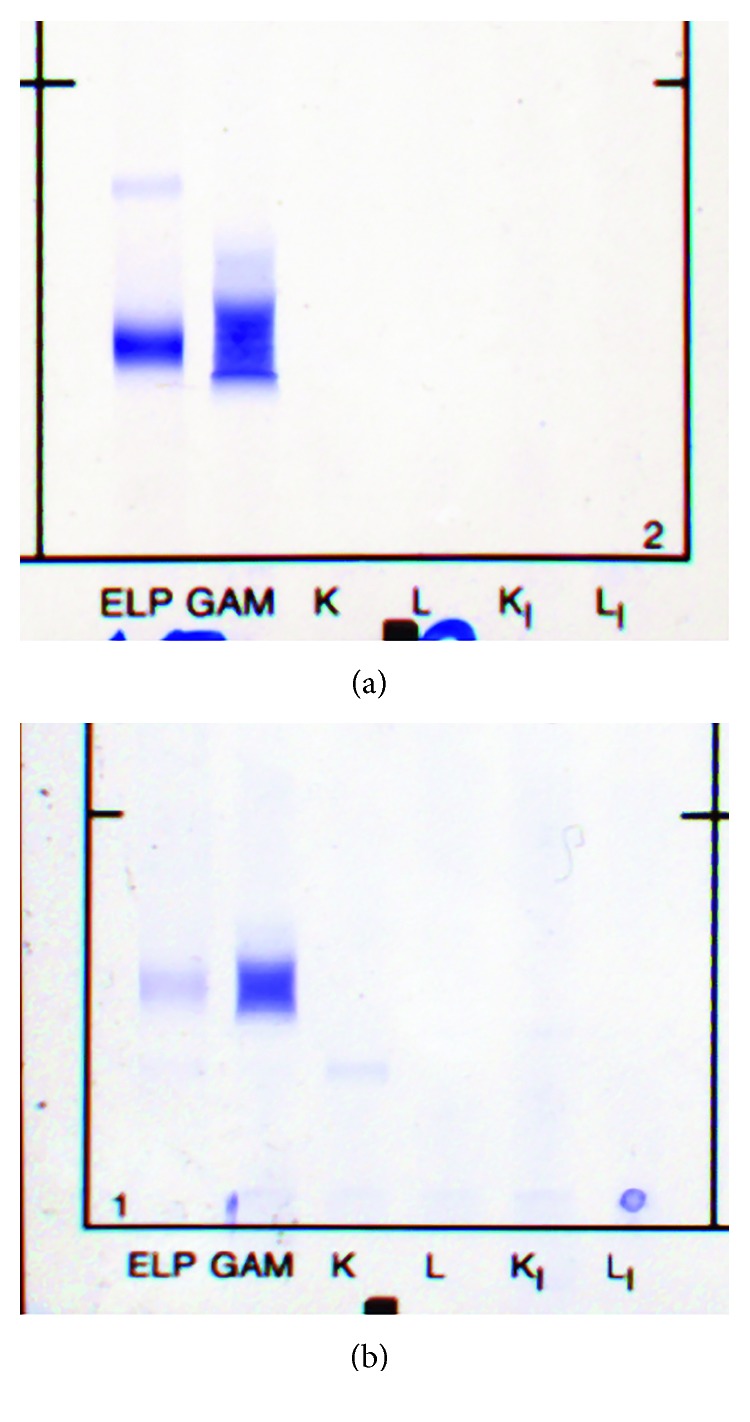
Urine electrophoresis and immunofixation. (a) Case 1: urine electrophoresis revealing a broad band and a minor band in the heavy chain region, without a corresponding band in the light chain region. (b) Case 2: urine electrophoresis revealing a broad band in the heavy chain region without a corresponding band in the light chain region. A further kappa light chain band is observed, corresponding to the IgMkappa band identified in the serum. ELP: reference track showing sample proteins; GAM: trivalent antiserum against gamma, alpha, and mu heavy chains; K and L: antisera against bound kappa and lambda light chains; K_I_ and L_I_: antisera against free kappa and free lambda light chains.

Gel protein electrophoresis and immunofixation (Figure 1(a)) and capillary zone electrophoresis and immunotyping (Figures 2(a) and 2(b)) (Sebia, UK) identified 2 *γ*-heavy chains. Both methods were negative for kappa and lambda light chains. Differing sensitivities of heavy and light chain reagents can cause false-negative results for light chain immunofixation, and the results were confirmed by a second gel electrophoresis (Helena, UK) method. Urine immunofixation identified a *γ*-heavy chain (Figure 3(a)).

Total IgG (determined by immunoturbidimetry on the Binding Site SPA-plus analyser) was elevated at 38.7 g/L (RR 7–16), with decreased levels of IgA and IgM (Table 1). IgG1 subclass levels were elevated, and other subclass levels were lower than the reference range (Table 3). Serum-free light chain ratios (determined by the Binding Site method, UK) were within the reference range (Table 4). HevyLite (Binding Site, UK) measurements are specific for individual heavy chains with either kappa or lambda light chains and can provide more specific information than individual immunoglobulin quantitation. Observed increased heavy chain pair ratios (e.g., IgMkappa/IgMlambda) can be indicative of clonal expansion. IgGkappa/IgGlambda ratios were within the reference range confirming the absence of clonal expansion of IgGkappa/IgGlambda intact immunoglobulins (Table 5).

In a previous study, HevyLite reagents which did not recognise intact IgG immunoglobulin ratios ((IgGkappa + IgGlambda)/total IgG) were used as indirect measures of heavy chain fragmentation [6]. The ratio of 0.04 in this patient was less than the average value of 0.18 found in the previous study [6].

**Figure 4:**
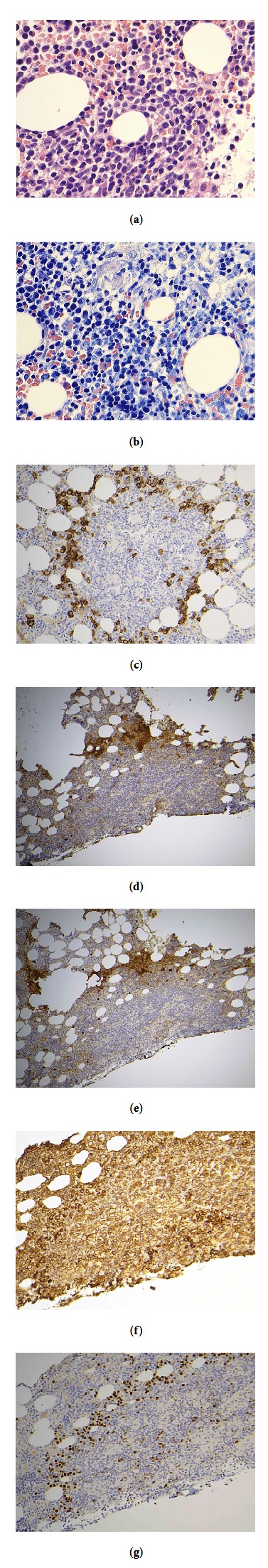
Bone marrow stain of Case 1. (a) Giemsa stain. (b) Small lymphocytes and scattered unusually small plasma cells. (c) CD138: plasma cells forming a characteristic corona around a lymphoid nodule. (d) Lambda stain—hardly any positive cells around the lymphoid nodule. (e) Kappa stain—hardly any positive cells around the lymphoid nodule. (f) IgG stain—a rim of strongly staining plasma cells. (g) Same area stained with MUM1 marker. Stains show there are IgG(+), kappa(−) and lambda (−) cells.

Bone marrow biopsy showed subtle infiltrates by low-grade B-NHL, associated with groups of clonal plasma cells, which expressed neither kappa nor lambda chain but stained with IgG heavy chain (Figures 4(a)–4(g)). Lymphatic infiltrate was surrounded by a rim of CD138+ plasma cells. Though there was equal expression of kappa and lambda light chains, the sum of kappa- and lambda-positive cells appeared to be much less than the number of CD138+ cells which indicated that most plasma cells do not express light chains.

The patient is currently monitored on a 3-monthly basis.

### 2.2. Case 2

**Figure 5:**
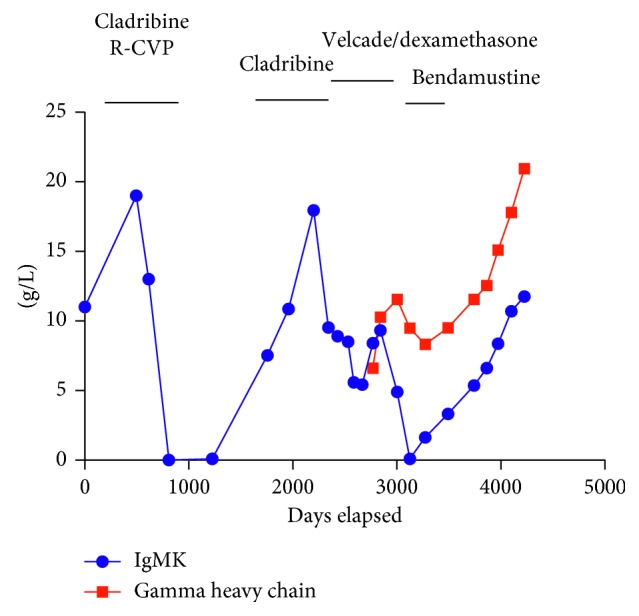
Paraprotein levels of Case 2 during treatment. Hospital records for the patient date back to time = 0 after chemotherapy. R-CVP = rituximab with cyclophosphamide, vincristine, and prednisolone.

**Figure 6:**
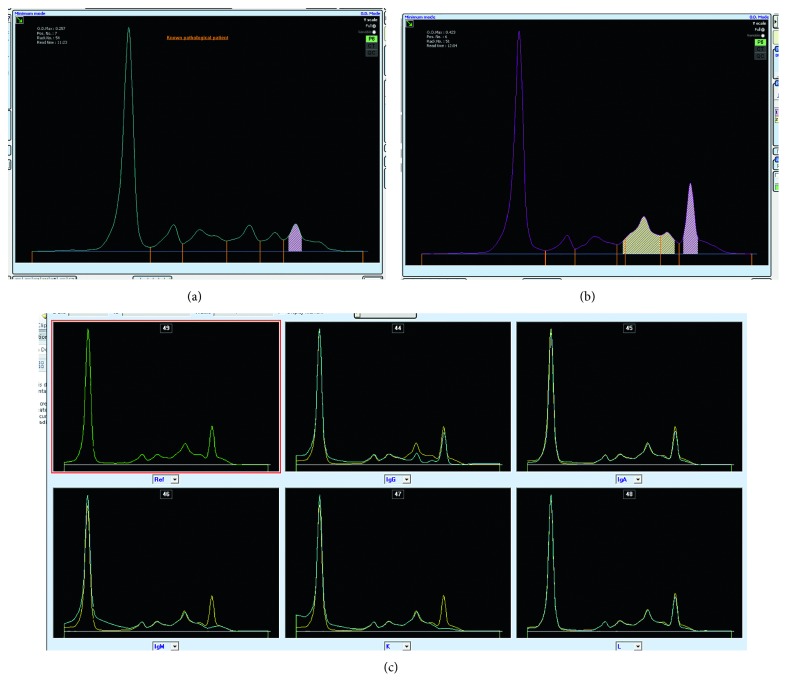
Capillary zone electrophoresis and immunotyping of Case 2. (a) Capillary zone electrophoresis prior to the appearance of the gamma heavy chain. A peak identified as the IgMkappa protein is detected in the gamma region. (b) Capillary zone electrophoresis identifies a broad peak in the beta region, identified as gamma heavy chain by immunotyping. (c) Immunotyping identifies a gamma heavy chain without a corresponding light chain and an IgMkappa paraprotein.

Patient 2 is an 81-year-old lady under treatment for WM (IgMkappa paraprotein). Details of her treatment are given in Figure 5. Capillary zone electrophoresis prior to the development of *γ*-HCD is shown in Figure 6(a). Gamma heavy chain developed during treatment and an unusually diffuse *γ*-heavy chain band was identified by gel electrophoresis (Figure 1(b)) and capillary zone electrophoresis (Figures 6(b) and 6(c)) (Sebia, UK) and confirmed by a second gel electrophoresis method (Helena, UK). Urine immunofixation identified the *γ*-heavy chain and kappa light chain (Figure 3(b)).

Her biochemistry and hematology prior to the development of *γ*-HCD and during follow-up are given in Tables 1 and 2. HevyLite measurements confirmed an IgMkappa paraprotein, and ratios of IgGkappa/IgGlambda were within the reference range, confirming increased IgG measurements were due to the IgG heavy chain (Table 5). The ratio of ((IgGkappa + IgGlambda)/total IgG) was 0.096.

The serum-free light chain ratio was elevated secondary to the presence of IgMkappa paraprotein (Table 4). Her total IgG levels were increased at 35.1 g/L, at diagnosis of *γ*-HCD, with suppressed IgA and an IgMkappa paraprotein of 10.7 g/L (Tables 1 and 2). IgG1 subtype was elevated, and other IgG subtypes were either decreased or at the lower reference range (Table 3). She is currently under observation for ibrutinib treatment.

**Figure 7:**
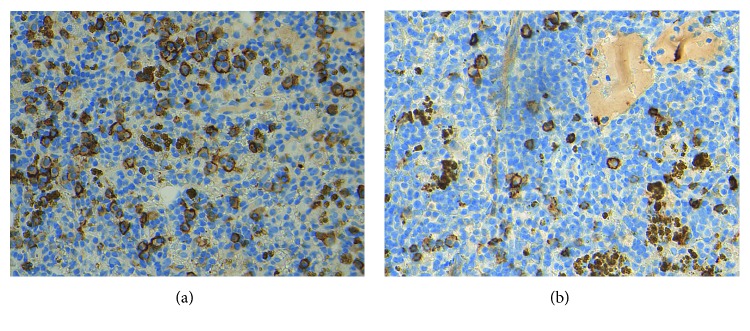
Bone marrow stain of Case 2. (a) CD138 highlights plasma cells—here admixed with lymphocytes (which are CD138 negative). Note also granular golden-brown haemosiderin deposits. (b) CD56—some plasma cells are positive; this is an interesting finding, as plasma cells of lymphoplasmacytic lymphoma usually do not express this marker. It indirectly suggests that the “other clone” may be more of plasma cell myeloma-like (myeloma-like MGUS). Haemosiderin which is naturally yellow/brown is present as well.

Her bone marrow results are shown in Figures 7(a) and 7(b) and in the Supplementary Material (available here). Lymphoma cells were CD5−, CD20+, and partially CD79a+, CD10+, CD23+, CD56+, and CD138+. A second CD56+ plasma cell clone most likely to be the *γ*-heavy chain producer was identified. Bone marrow histology identified lymphoplasmacytic lymphoma/WM, which appeared as IgM/kappa plus another population of scattered plasma cells, occasionally expressing IgG and/or lambda.

## 3. Discussion

Heavy chain diseases are rare B-cell lymphoproliferative disorders that are characterised by the production of immunoglobulin heavy chains without associated light chains. *γ*-HCD has a variety of clinical presentations. The Mayo Clinic reported a series of 23 patients of whom 70% had an underlying lymphoplasma cell proliferative disorder, 13% an autoimmune disorder, and 4% no underlying disease. Pathological diagnosis of underlying disease reported in the 23 patients was lymphoplasma cell proliferative disorder, lymphoma, chronic lymphocytic leukemia, plasmacytoma, and plasma cell proliferative disorder. The disease was associated with lymphadenopathy (34%), hepatomegaly (4%), splenomegaly (30%), or bone marrow involvement (30%). The median survival of the 23 patients was 7.4 years (range 1 month to more than 21 years) [7]. In a further series of 13 patients, 8 patients showed a lymphoplasmacytic neoplasm that was difficult to classify. In the remaining 5 patients, biopsy identified well-defined neoplasms: splenic diffuse red pulp and small B-cell lymphoma, splenic marginal zone lymphoma, MALT lymphoma, lymphoplasmacytic lymphoma/WM, and chronic NK cell lymphocytosis. In the 13 cases, the study reported the involvement of the lymph nodes (*n*=7), spleen (*n*=2), bone marrow (*n*=8), and other extranodal tissues (*n*=3). Patients showed a female predominance (85%) with frequent occurrence of autoimmune disease (69%) [8]. Iijima et al. [9] report a case *γ*-HCD and T-cell large granular lymphocytic leukemia. *γ*-HCD can exist in the absence of clinical symptoms similar to other forms of monoclonal gammopathy of undefined significance (MGUS) [10].

In our patients, two methods, capillary zone electrophoresis and gel electrophoresis, identified a gamma heavy chain with no corresponding light chain. Results from this report and previous reports [11] suggest that capillary zone electrophoresis and immunotyping are able to detect and characterise *γ*-HCD in serum. *γ*-Heavy chain was identified in the urine of both patients, suggesting that the lower-molecular weight of the truncated heavy chain allowed renal clearance. In a modification of the immunofixation method, Gulli et al. [12] preincubated patient sera with anti-kappa and anti-lambda to precipitate intact immunoglobulins, except for free heavy chain, prior to immunofixation.

The intact IgGkappa/IgGlambda (HevyLite) measurement confirmed the presence of gamma heavy chain in the absence of associated kappa or lambda light chains. In a previous study, HevyLite assays showed that in 10/15 patients, substantial portions of IgG were not quantitated by antibodies to intact IgGkappa and IgGlambda immunoglobulins [6].

Serum IgG subtypes showed an increase in IgG1 subclass in both patients. In a previous report, serum IgG measured by nephelometry on the Beckman Coulter Immage analyser was below the reference range in a patient with *γ*-HCD [11]. Total IgG and IgG1 subclasses measured using the binding site reagent were increased in our patients. This has been previously illustrated by other groups [13]. The discrepant values observed between case reports can be the result of differences in analytical techniques, loss of antigenic domains due to truncation of the heavy chains, or analytical variation due to different detection antibody specificities.

There have been several advances in the diagnosis of multiple myeloma. New techniques may further enhance the diagnosis of *γ*-HCD. Capillary electrophoresis with immunotyping is able to characterise free heavy chains in serum, demonstrating the absence of intact IgG immunoglobulins by the HevyLite assay and the absence of serum-free light chains by serum-free light analysis are further valuable tools for diagnosis. Serum/urine electrophoresis and immunofixation are analytical tools in the workup of *γ*-HCD patients.

Alpha (*α*), *γ*, and mu (*µ*) heavy chain diseases have been described. *α*-HCD, the most common of heavy chain diseases, appears to be a condition affecting primarily the secretory IgA system and mainly the digestive tract. *α*-HCD may present as a continuous sequence of events from an apparently reversible hyperplastic process to an overt neoplastic proliferation [14]. *µ*-HCD is the least common of the heavy chain diseases. Patients present with lymphoplasma cell proliferative disorder, and index of suspicion is high in patients with vacuolated plasma cells in the bone marrow [3].

The mechanism for the pathogenesis of *γ*-HCD is unclear. Bone marrow histology of the first patient with isolated *γ*-HCD suggested the presence of a clonal plasma cell which selectively secreted *γ*-heavy chain in the absence of kappa or lambda light chain. Genomic events that underlie the progression of MGUS to MM have been difficult to dissect. Both bone marrow microenvironment and genomic instability play a role in MM pathogenesis. Bone marrow biopsy in the second patient, with WM and *γ*-HCD, identified two possible malignant clones. It is worth noting that this patient's treatment included R-CVP, cladribine, and velcade. It is plausible that the treatment might have increased the transformation of the *γ*-HCD clone by increasing the instability of the MM cells. Clonal evolution in MM before and after therapy can follow several patterns. Branching clonal evolution involves one or more subclones that emerge at a later time point. Current therapeutic approaches have shortcomings, and understanding of MM tumorogenesis and progression may have implications for genomically guided precision treatment [15].

## 4. Conclusions

In summary, we report two cases of *γ*-HCD. The findings also provide information for practical guidance for the laboratory evaluation of *γ*-HCD. In the first case, *γ*-HCD developed as the presenting lymphoid malignancy. Bone marrow biopsy revealed an IgG heavy chain secreting clone. The second case was a composite lymphoid neoplasm of WM and *γ*-HCD. *γ*-HCD developed during treatment for WM. Bone marrow biopsy revealed the presence of two neoplastic lymphoid cell lines. The review of the literature suggests further studies are indicated to develop an understanding of the clonal evolution of *γ*-HCD.

## Figures and Tables

**Table 1:** Hematology and biochemistry results.

Date	Hb, g/L (RR 115–164)	WBC, 10^9^/L (4–11)	Platelet, 10^9^/L (150–400)	Neutrophils, 10^9^/L (2–7.5)	Creatinine, *µ*mol/L (RR 44–80)	Calcium, mmol/L (RR 2.2–2.6)
*Case 1*						
At diagnosis	115	2.5	100	1.6	46	2.41
3 months following diagnosis	116	2.8	101	2.1	61	2.33

*Case 2*						
Postcladribine at time 0 days	120	5.0	239	3.1	71	
At 10-year follow-up and treatment	89	3.4	133	2.0	65	2.41

Case 2: records date back to time = 0 after chemotherapy.

**Table 2:** Monoclonal protein studies.

Date	Total protein, g/L (RR 60–80)	Albumin, g/L (RR 35–50)	Monoclonal band 1, g/L	Monoclonal band 2, g/L	IgG, g/L (RR 7–16)	IgA, g/L (RR 0.7–4.0)	IgM (RR 0.4–2.3)
*Case 1*							
At diagnosis	60	33	2.8 (type: y-heavy chain in the alpha region)	18.5 (y-heavy chain in the beta region)	38.7	0.19	0.26
3 months following diagnosis	63	33	3.21	21.17	39.7	0.15	0.24

*Case 2*							
At time 0	78	39	11.0 (IgMK)		11.6	0.8	
At 10-year follow-up, after several treatments	76	40	10.7 (IgMK)	17.8 (*γ*-heavy chain)	35.1	0.06	

Case 2: records on the patient date back to time = 0 after chemotherapy.

**Table 3:** IgG subclasses.

	Case 1	Case 2
IgG1 (RR 3.8–9.29 g/L)	18.62	27.16
IgG2 (RR 2.42–7.0 g/L)	0.26	2.92
IgG3 (RR 0.22–1.76 g/L)	0.41	0.17
IgG4 (0.04–0.86 g/L)	<0.03	0.12

**Table 4:** Serum-free light chains.

Light chain type	Case 1	Case 2 (at time 0)
Kappa (3.3–19.4 mg/L)	18.47	33.94
Lambda 16.60 mg/L (5.7–26.3 mg/L)	16.8	18.77
Kappa/lambda ratio (0.26–1.65)	1.113	1.808

**Table 5:** HevyLite protein studies.

Heavy chain type	Case 1	Case 2
IgGkappa (3.84–12.07 g/L)	0.99	1.92
IgGlambda (1.91–6.74 g/L)	0.54	1.45
IgG K/L ratio (1.12–3.21)	1.83	1.32
IgA K (0.57–2.08 g/L)	0.23	<0.02
IgAL (0.44–2.04 g/L)	0.12	0.03
IgA K/L ratio (0.78–1.94)	1.92	Not available
IgMK = 0.27 g/L (0.19–1.63 g/L)	0.27	11.92
IgML = 0.04 g/L (0.12–1.01 g/L)	0.04	0.14
IgM K/L (1.18–2.74)	6.75	85.1

## Supplementary Materials

Supplementary Materials Bone marrow stain of Case 2, with anti-kappa and anti-lambda, is shown in Figures 8(a) and 8(b).
