# Supplementary material for: Two Cases of γ-Heavy Chain Disease and a Review of the Literature
Source: Case Rep Hematol. 2018 Aug 12;2018:4832619. doi: 10.1155/2018/4832619 (PMC6109557; doi:10.1155/2018/4832619)
Supplement: Supplementary Materials — Bone marrow stain of Case 2, with anti-kappa and anti-lambda, is shown in Figures 8(a) and 8(b). [file 4832619.f1.pdf]

SUPPLEMENTARY MATERIAL

Case 2: Bone Marrow

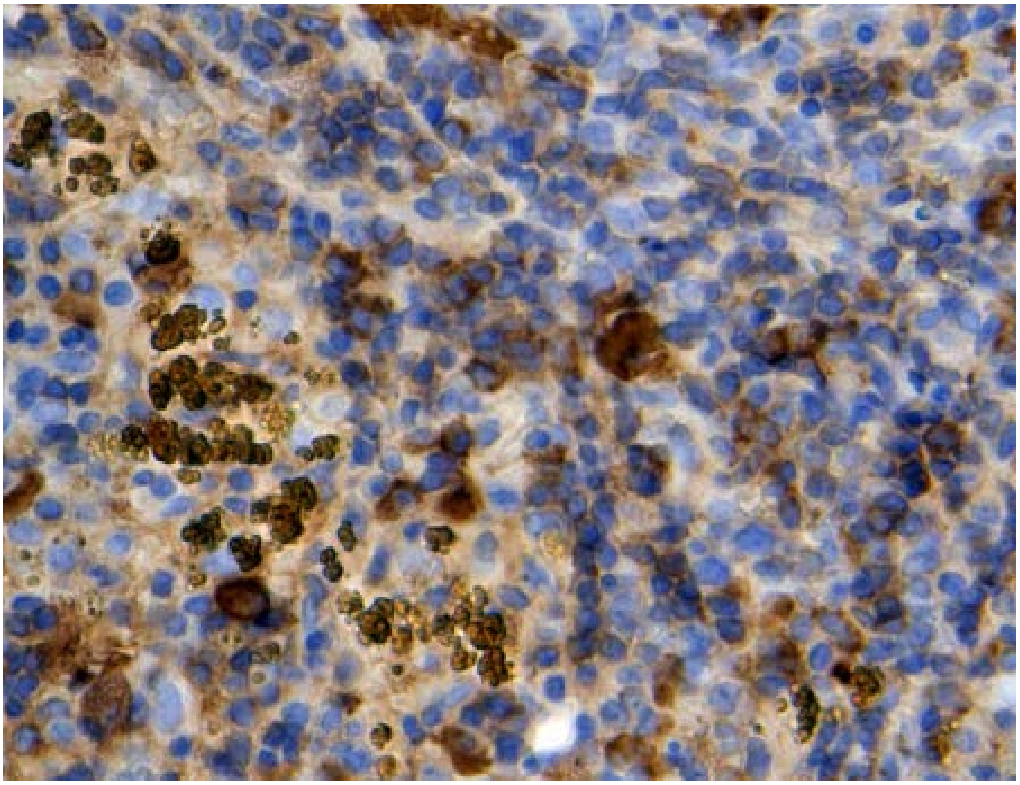

kappa stain - hint of expression on lymphocytes, a few plasma cells strongly positive

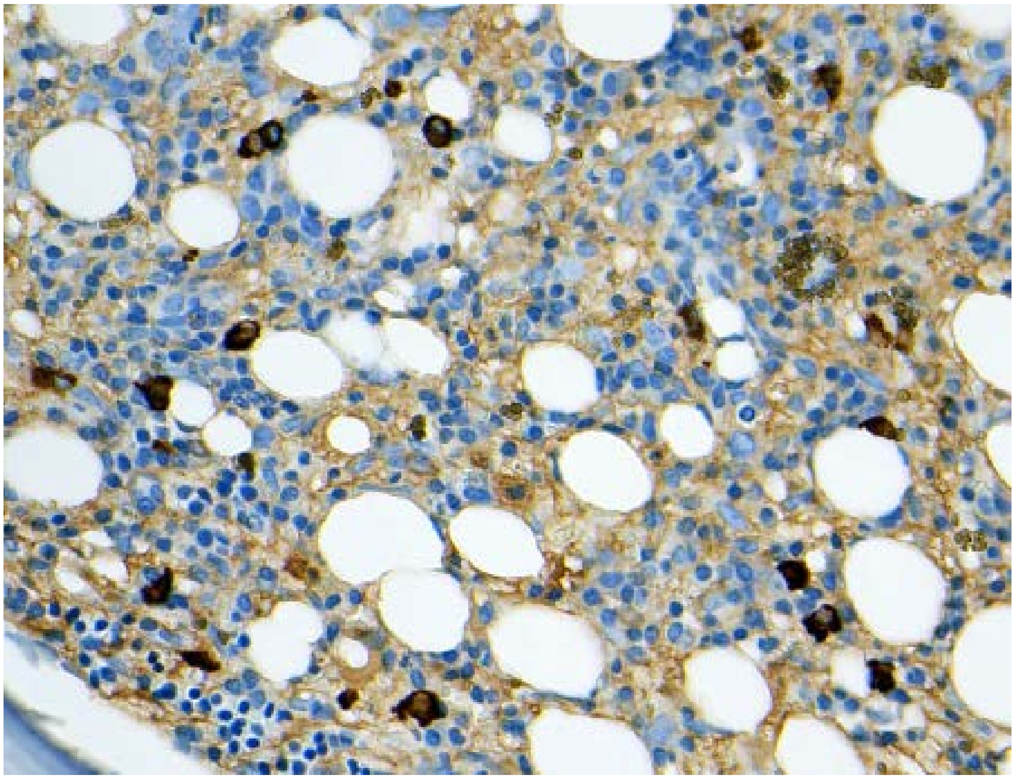

lambda stain – some plasma cells strongly positive but lymphocytes appear negative
